# Innate immune recognition and microenvironmental reprogramming in HPV-induced cervical cancer: from pattern recognition receptor activation to immune tolerance disruption

**DOI:** 10.3389/fcimb.2026.1758873

**Published:** 2026-04-07

**Authors:** Shahid Ullah Khan, Mustafa H. Halawi, Mazen Almehmadi, Ramadan Taha, Ahmed Ezzat Ahmed, Mohammad Y. Alfaifi, Ali A. Shati, Serag Eldin I. Elbehairi, Saleem Ahmad, Yasmene F. Alanazi, Mohammed Al-Rasheed, Nuruliarizki Shinta Pandupuspitasari, Endang Widiastuti, Munir Ullah Khan

**Affiliations:** 1 Department of Biomedical Sciences, College of Medicine, Dubai Medical University (COM, DMU), Dubai, United Arab Emirates; 2 Department of Animal Science, Faculty of Animal and Agricultural Sciences, Universitas Diponegoro. Jl. Prof. Jacub Rais, Semarang, Central Java, Indonesia; 3 Medical Laboratory Technology, College of Nursing and Health Sciences, Jazan University, Jizan, Saudi Arabia; 4 Department of Clinical Laboratory Sciences, College of Applied Medical Sciences, Taif University, Taif, Saudi Arabia; 5 Biology Department, Faculty of Science, King Khalid University, Abha, Saudi Arabia; 6 Prince Sultan Bin Abdelaziz for Environmental Research and Natural Resources Sustainability Center, King Khalid University, Abha, Saudi Arabia; 7 Department of Cell Biology and Physiology, University of Kansas Medical Center, Kansas, KS, United States; 8 Department of Biochemistry, Faculty of Science, University of Tabuk, Tabuk, Saudi Arabia; 9 Department of Clinical Sciences, College of Veterinary Medicine, King Faisal University, Al-Ahsa, Saudi Arabia; 10 Food Research for Safety, Security, and Sustainability (FORC3S), Semarang, Indonesia; 11 Faculty of Animal and Agricultural Sciences, Universitas Diponegoro, Semarang, Central Java, Indonesia; 12 Department of Pharmacy, IQRA University Chak, Islamabad, Pakistan

**Keywords:** HPV, immune tolerance, immunity in the female genital tract, innate immune responses in the reproductive system, microenvironment at the cervical region, PRRs, TLRs

## Abstract

Innate immune recognition plays a central role in determining the outcome of human papillomavirus (HPV) infection and the subsequent development of cervical cancer. This mini-review highlights how the reproductive tract’s innate immune system, particularly Pattern Recognition Receptors (PRRs) such as Toll-like receptors (TLRs), NOD-like receptors (NLRs), and RIG-I-like receptors (RLRs), detects HPV-associated molecular patterns and initiates antiviral defenses. HPV has evolved sophisticated strategies to evade these responses by suppressing PRR signaling, altering cytokine networks, reprogramming cellular metabolism, and reshaping the cervical microenvironment. These viral mechanisms contribute to the formation of a persistent post-infection microenvironment (PIM), characterized by impaired antigen presentation, regulatory immune cell infiltration, chronic inflammation, and metabolic and stromal remodeling, which collectively promote immune tolerance and carcinogenesis. Emerging evidence also highlights the roles of inflammasomes, type I interferon pathways, and extracellular vesicles in modulating innate immune responses during HPV infection. Understanding how innate immunity senses HPV and how the virus circumvents these pathways provides crucial insight into cervical cancer progression and offers opportunities for developing more effective immunotherapies, vaccines, and prevention strategies. This review synthesizes current advances in HPV-driven innate immune dysregulation within the reproductive tract and their implications for reproductive immunology and infection-associated malignancy.

## 1 Introduction

Human papillomavirus (HPV) infection is the primary etiological driver of cervical cancer, a significant cause of morbidity and mortality in women worldwide (Sung et al., 2021). While most HPV infections are transient and cleared by host immunity, persistent infection with high-risk genotypes reflects a failure of innate immune recognition and the establishment of a permissive microenvironment that enables viral persistence and malignant progression. The cervical epithelium relies on Pattern Recognition Receptors (PRRs), including Toll-like receptors (TLRs), NOD-like receptors (NLRs), and RIG-I-like receptors (RLRs), to detect viral components and initiate antiviral and inflammatory responses (Bogani et al., 2022; Guimarães et al., 2022). However, HPV oncoproteins E5, E6, and E7 strategically suppress PRR signaling, impede interferon pathways, reduce antigen presentation, and disrupt epithelial–immune communication (Cho and Chun, 2018). These immune-evasive mechanisms contribute to the development of a post-infection microenvironment (PIM) characterized by immunosuppression, metabolic reprogramming, altered stromal signaling, and impaired cytotoxic lymphocyte activity (Perkins et al., 2023). For patients reporting locally advanced disease, recurrence, or metastatic spread of malignant tumors, although there has been a significant improvement in the treatment of cervical cancer, clinical results still show minimal improvement. The latest advancements in cancer immunobiology have clarified the fundamental process by which cancer cells evade immune surveillance, making immunotherapy a revolutionary treatment approach for cervical cancer, especially in advanced cases cytotoxic chemotherapy and radiation therapy and surgical removal of the traditional treatment interventions on the contrary, such as immune treatment technology by enhancing the body’s natural antitumor immune response, thus provides a new treatment possibilities (Xu et al., 2025).

This Mini review first examines innate immune sensing pathways involved in HPV recognition, including TLR-, RIG-I-, and cGAS-STING-mediated signaling. We then discuss how HPV oncoproteins disrupt these pathways, establishing a post-infection microenvironment characterized by immune suppression and metabolic reprogramming. Finally, we highlight therapeutic strategies to restore antiviral immunity through multimodal interventions.

## 2 Immune microenvironment in cervical cancer

Human papillomavirus (HPV)-related cervical cancer exhibits unique immunological characteristics in its microenvironment (Kudela et al., 2021). After viral infection, keratinocytes strategically alter the environment to prevent effective immune-mediated viral eradication, while also forming complex communication networks with host stromal components. The supportive niches created by these interrelated processes have promoted prolonged viral infection, increased viral transmission, and the progression of cervical malignancy (Yuan et al., 2021). Pathological studies have shown that tumor-associated macrophages (TAMs), especially those expressing CD68+ and CD163+ markers, exhibit a progressive infiltration pattern, ranging from histologically normal cervical epithelium to progressively severe cervical intraepithelial neoplasia (CIN) grades I-III, and then to direct invasive cervical cancer. According to morphometric research, a higher FIGO disease stage and lymph node metastasis are closely associated with increased CD163+ TAM infiltration. These immune cell subsets serve as useful prognostic indicators of tumor progression and metastatic potential.

Functional repolarization of immunosuppressive phenotypes, characterized by impaired antigen presentation, inhibition of active T cell proliferation, and increased angiogenic activity, occurs in response to tumor-derived signals in TAMs. These phenotypes act synergistically to increase the potential for tumor invasion and metastatic spread (Chen et al., 2017). Significantly, HPV oncoproteins E6 and E7 directly induce immunosuppression in the tumor microenvironment through multiple molecular pathways. In particular, by stimulating the PI3K/Akt pathway, the E6 viral oncoprotein promotes enhanced expression of programmed death-ligand 1 (PD-L1), thereby stabilizing hypoxia-inducible factor-1α (HIF-1α) and subsequently amplifying PD-L1 transcription (Konstantopoulos et al., 2024). Meanwhile, the E7 viral oncoprotein activates the signal transducer and the activator of transcription 3 (STAT3) pathway, thereby promoting T cell dysfunction and increasing PD-L1 expression. These coordinated molecular processes enable HPV-transformed cells to evade immune detection and inhibit the cytotoxic T lymphocyte-dependent clearance system (Li Y. et al., 2025).

**Figure 1:**
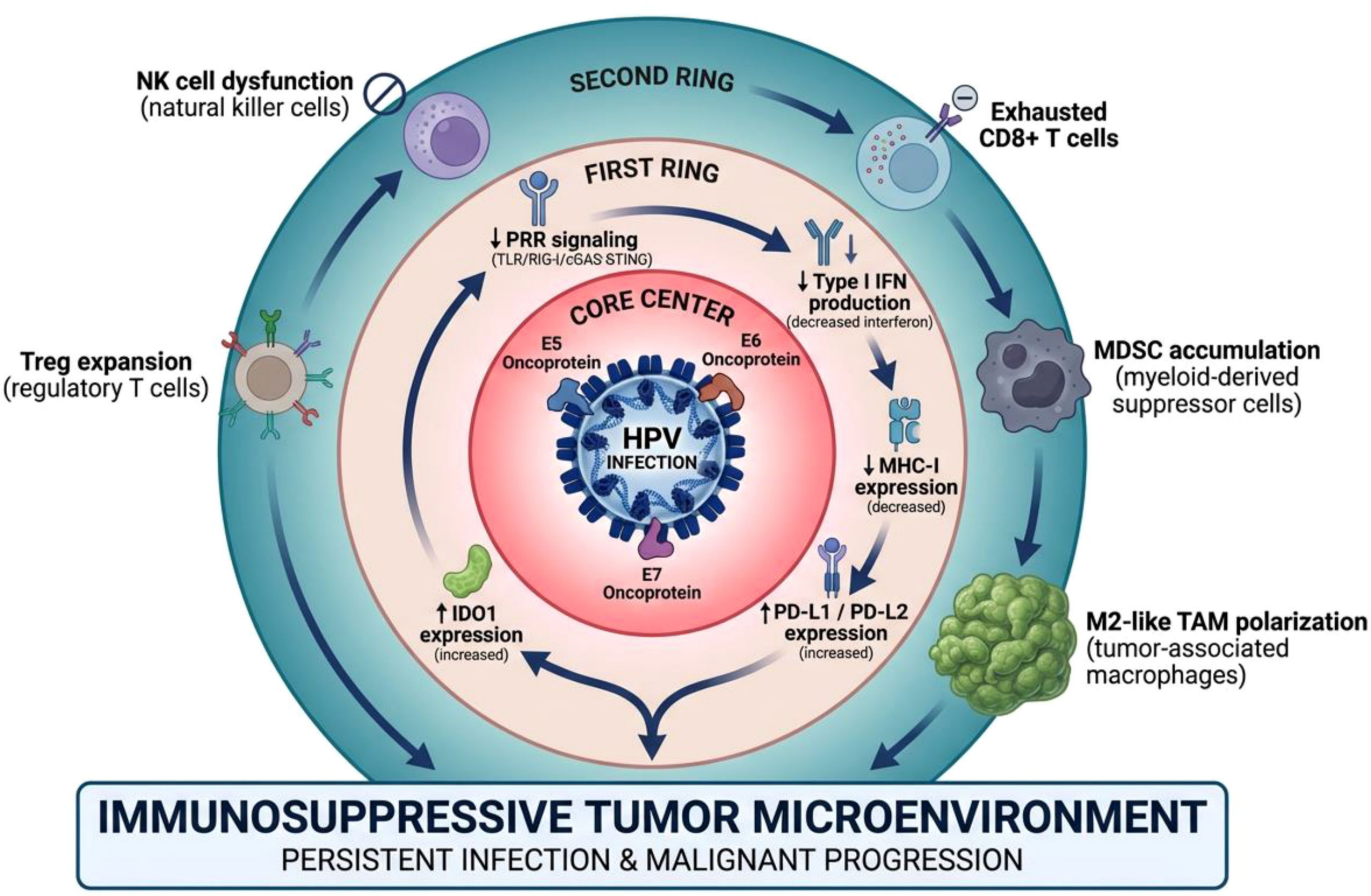
HPV-mediated immune suppression in the cervical cancer microenvironment.

The effector activity of natural killer (NK) cells is simultaneously impaired by persistent HPV infection (Zhang et al., 2019). Cervical tumor lesions are characterized by significant infiltration of CD8+ T lymphocytes. However, these cytotoxic cells cannot effectively control the expansion of malignant cells, possibly due to impaired cell lysis and weakened HPV-mediated immune surveillance, resulting in a predominantly immunosuppressive tumor microenvironment (TME) (Wang et al., 2018). HPV oncoprotein causes extensive alterations in metabolic pathways, significantly influencing immune cell activity beyond the checkpoint inhibition mechanism (Huang et al., 2024). It is worth noting that the E7 oncoprotein exerts an immunosuppressive effect by activating the nuclear factor-κB (NF-κB) signaling cascade, thereby increasing indoleamine 2,3-dioxygenase 1 (IDO1) expression, which leads to increased tryptophan degradation and the accumulation of kynurenine metabolites. To maintain immune tolerance, the consumption of tryptophan substrates generates metabolic constraints, preventing the proliferation of effector T cells. At the same time, the accumulation of kynurenine promotes the formation of regulatory T cell populations (Kashyap et al., 2024). The interferon-γ (IFN-γ) signaling pathway is often overactivated in long-term HPV infection. It works in concert with E6/E7 viral oncoproteins to further increase IDO1 expression, thereby enhancing immunosuppression of the tumor microenvironment. These molecular research results demonstrate how important it is to use treatment methods targeting HPV-mediated immune dysregulation to improve patient prognosis, especially when it comes to recurrent and disseminated cervical cancer (**Figure 1**).

## 3 Formation and components of PIM

In this review, we define the post-infection microenvironment (PIM) as a distinct, HPV-conditioned immune niche that emerges before malignant transformation and is driven by persistent viral infection rather than tumor-intrinsic genetic instability. Unlike the classical tumor microenvironment (TME), which develops after oncogenic transformation and is shaped mainly by cancer cell metabolism, hypoxia, and genomic alterations, the PIM is established through long-term expression of HPV early genes (E5, E6, and E7), chronic low-grade inflammation, and sustained interference with innate immune sensing pathways (Zhang et al., 2025). Persistent HPV infection reprograms epithelial–immune crosstalk by suppressing PRR-mediated interferon responses, altering cytokine and chemokine gradients, and promoting tolerogenic antigen presentation, thereby creating an immune-permissive niche that facilitates viral persistence and sets the stage for subsequent carcinogenesis (Ferreira et al., 2020).

PIM formation is initiated when HPV viral particles enter keratinocytes in the basal layer of the cervical stratified squamous epithelium (Sung et al., 2021). This process may be precipitated by mechanical trauma, inflammation, hyperthermia, or radiation-induced damage (Wendel and Wallace, 2023). The coordinated interaction between HPV-infected keratinocytes and immune cells, along with host matrix components and their derivatives (including chemokines, cytokines, and metabolites), orchestrates PIM formation. Actively suppresses keratinocyte innate immune responses by inhibiting.

In addition to protein-level immune evasion, HPV modulates host immunity by splicing viral transcripts. A recent study demonstrated that a high unspliced-to-spliced E6 transcript ratio (E6 ratio) is significantly associated with reduced infiltration of key effector immune populations, including activated dendritic cells (aDCs), M1 macrophages, and natural killer T (NKT) cells, in cervical cancer tissues (Sasagawa et al., 2012). This finding establishes a direct link between HPV E6 splicing balance and immune microenvironment remodeling, indicating that post-transcriptional regulation of viral oncogene expression contributes to immune tolerance during persistent HPV infection.

Cervical keratinocytes detect HPV through multiple Pattern Recognition Receptors (PRRs). Endosomal TLRs, particularly TLR3 and TLR9, recognize viral nucleic acids and activate TRIF- or MyD88-dependent pathways, resulting in NF-κB and IRF3/7 activation and type I interferon production. Cytosolic RIG-I-like receptors (RIG-I and MDA5) signal via MAVS to amplify antiviral responses. In parallel, NOD-like receptors such as NLRP3 contribute to inflammasome activation and IL-1β maturation. Persistent HPV infection disrupts these coordinated sensing mechanisms, thereby attenuating antiviral immunity and facilitating immune tolerance. Keratinocytes express various pathogen recognition receptors, including Toll-like receptors (TLRs) and retinoic acid-inducible gene I (RIG-I) receptors, functioning as innate immune sentinels with antiviral capabilities (Rattay et al., 2022). However, HPV employs sophisticated mechanisms to interfere with multiple keratinocyte signaling pathways and evade epithelial immune surveillance. Upon nuclear entry, HPV expresses early oncoproteins E1 and E2 to maintain low-level viral genome amplification, while E5, E6, and E7 proteins actively suppress keratinocyte innate immune responses (James et al., 2020).

**Figure 2:**
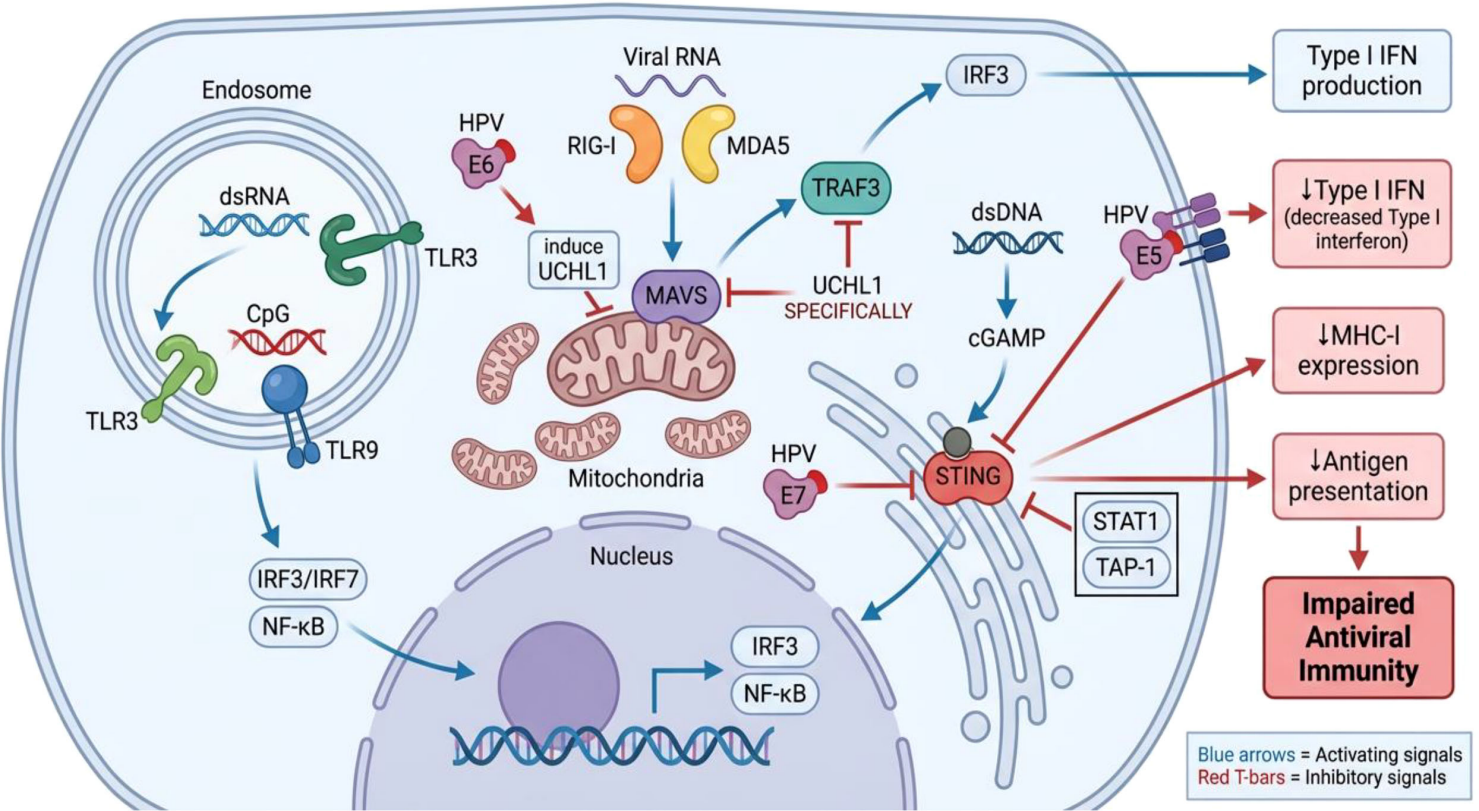
HPV-mediated inhibition of innate immune sensing pathways in cervical keratinocytes.

Mechanistically, HPV oncoproteins target multiple nodes of PRR-driven antiviral signaling. E6 and E7 induce the host deubiquitinase UCHL1, which restricts TRAF3 activation and impairs IRF3 dimer nuclear translocation, thereby suppressing type I interferon induction. In addition, HPV18 E7 antagonizes cGAS STING signaling through a direct interaction with STING, selectively inhibiting cGAS-STING-driven NF-κB activation (including reduced nuclear accumulation of p65) (Sun et al., 2025). HPV E5 further suppresses DNA-sensing pathways by directly interacting with STING to blunt downstream interferon signaling, and has been linked to reduced immunoproteasome function and altered antigenic peptide presentation, collectively diminishing immune detection and immunotherapy responsiveness. The HPV E5 protein constrains the antigen repertoire presented by major histocompatibility complex (MHC) molecules on the cell surface (Miyauchi et al., 2023). HPV E6 and E7 proteins induce ubiquitin carboxy-terminal hydrolase L1 (UCHL1), which restricts tumor necrosis factor receptor-associated factor 3 (TRAF3) activation and inhibits nuclear translocation of interferon regulatory factor 3 (IRF3) dimers, consequently suppressing interferon expression (Karim et al., 2013). The HPV18 E7 protein specifically inhibits the cyclic GMP-AMP synthase (cGAS)-stimulator of interferon genes (STING) signaling pathway (Lou et al., 2023). Similarly, the HPV16 E7 protein blocks interferon-γ-induced signal transducer and activator of transcription 1 (STAT1) phosphorylation, suppressing IRF-1 and transporter associated with antigen processing subunit 1 (TAP-1) expression, thereby reducing the efficiency of MHC class I antigen presentation on keratinocyte surfaces (Kletschkus et al., 2020). Collectively, these pathways facilitate PIM formation by altering keratinocyte characteristics and disrupting immune mediators (**Figure 2**).

## 4 Immunosuppressive microenvironment promotes persistent HPV infection

Neoantigens generated by oncogene overexpression are released following cell death, captured and presented to T lymphocytes by APCs, activating effector T cells to eliminate cancer cells and release additional antigens. This cyclical process defines the cancer-immune cycle. However, in high-grade cervical lesions, tumor-associated macrophage (CD163+CD68+) infiltration increases while interferon-γ production decreases (Chen et al., 2017). In infected keratinocytes, reduced interferon levels, downregulation of adhesion molecules, restricted viral epitope presentation, and decreased chemokine production result in diminished APC recruitment at infection sites. This impairs recognition and clearance of virus-infected cells. Additionally, effector T lymphocytes undergo exhaustion, facilitating immune escape by cervical cancer cells (Chen et al., 2023). Immune cells migrate to inflammatory sites, where they release cytokines and reactive oxygen species, thereby disrupting cellular homeostasis. Compared to healthy populations, cervical cancer patient sera exhibit elevated expression of cyclooxygenase-2 (COX-2), soluble triggering receptor expressed on myeloid cells-1 (sTREM-1), β-interferon, IL-1β, and IL-6 (Syahruddin et al., 2025). Further studies demonstrate that HPV E5, E6, and E7 proteins increase COX-2 expression via the activator protein-1 (AP-1) pathway, leading to the release of substantial prostaglandins with detrimental effects on cervical tissue (Hemmat and Bannazadeh Baghi, 2019). Conversely, oxidative stimulation may perpetuate inflammatory responses, cause DNA damage and genomic instability, and create conditions favorable for HPV DNA integration.

HPV16 E6 protein upregulates hypoxia-inducible factor-1α (HIF-1α) and promotes VEGF expression, demonstrating potent angiogenic activity (Rho et al., 2020). Vascular basement membrane degradation by matrix metalloproteinase (MMP) proteolytic activity, particularly MMP-2 and MMP-9, exposes VEGF-A receptors on vascular endothelial cells (Azevedo Martins et al., 2020), promoting further angiogenesis. Tumor cells enter the bloodstream through spaces created by the rupture of the vascular basement membrane, enabling distant metastasis. HPV18 E6 and E7 proteins enhance hexokinase 2 (HK2) transcription, the rate-limiting enzyme in glycolysis, playing crucial roles in the aerobic glycolysis of cervical cancer cells (Prakasam et al., 2022). E6 protein inactivates p53 and directly inhibits miRNA-34a (miR-34a) expression. Reduced miR-34a promotes the Warburg effect, thereby increasing adenosine triphosphate production (Zhang et al., 2016).

## 5 Therapeutic strategies targeting PIM

Immunotherapeutic strategies targeting immune cells within the PIM can achieve precise HPV infection prevention and control, ultimately leading to cervical cancer prevention and control. Cervical cancer immunotherapy comprises four primary categories: immune checkpoint blockade, adoptive cell transfer, therapeutic vaccines, and cytokine therapy. Programmed cell death protein-1 (PD-1) and cytotoxic T lymphocyte-associated antigen-4 (CTLA-4) represent promising therapeutic targets for cervical cancer with significant clinical potential.

Clinical investigations have evaluated PD-1 checkpoint inhibitors, including nivolumab and pembrolizumab, as well as programmed cell death ligand-1 (PD-L1) checkpoint inhibitors, such as atezolizumab and durvalumab. These studies demonstrate that PD-1/PD-L1 blockers can increase CD8+ forkhead box protein 3 (FoxP3)+ CD25+ T lymphocyte subsets in patients with cervical cancer, as shown in **Table 1**, while reducing immunosuppressive regulatory T lymphocytes (Heeren et al., 2019). Following treatment with engineered T lymphocytes targeting HPV16 E7 protein (NCT02858310), tumor regression occurred in 6 of 12 HPV16-positive cervical cancer patients, including 4 cases with anti-PD-1 refractory tumors (Nagarsheth et al., 2021). Therapeutic DNA vaccines incorporating C-C motif chemokine ligand 11 (CCL11) fused to E6 and E7 antigens can expand T lymphocyte receptor diversity and induce infiltration of activated T cells, macrophages, and DCs into tumors, thereby establishing an anti-tumor immune microenvironment (Qi et al., 2024). Although various methods are summarized in **Table 1**, the clinical response to single immunotherapy in HPV-related cervical cancer remains limited, reflecting the existing immune barrier. Persistent HPV infection promotes early immune tolerance by inhibiting PRR signaling, the interferon response, and antigen presentation, thereby creating an inflammatory and immunosuppressive environment that precedes tumor development. Other barriers include HPV-mediated downregulation of MHC class I, enrichment of immunosuppressive cells (such as regulatory T cells and m2 macrophages), and checkpoint redundancy, all of which lead to T cell failure. The curative effect of these standards limits the monotherapy (Jiang et al., 2022). These features aim to restore innate awareness, enhance antigen presentation, and mitigate the combined impact of adaptive immune suppression strategies, thereby providing a robust immunological foundation.

**Table 1:** Current and Emerging Therapeutic Strategies for HPV-Positive Cervical Cancer.

Therapeutic category	Agent/approach	Target/mechanism	Clinical status	Key findings/efficacy	Limitations	References
Immune Checkpoint Blockade	Pembrolizumab	PD-1 inhibition	Phase II approved	Increased CD8+FoxP3+CD25+ T cells	Variable response rates	(Tewari et al., 2024)
	Nivolumab	PD-1 inhibition	Phase II trials	33% ORR in HPV16+ patients	Treatment-related toxicity	(Xie et al., 2022)
	Atezolizumab/ Durvalumab	PD-L1 inhibition	Clinical trials	Reduced Tregs, enhanced cytotoxicity	Limited long-term data	(Liu et al., 2025)
Therapeutic Vaccines	ISA101	HPV16 E6/E7 peptides	Phase II	Lesion clearance in VIN	Ineffective as monotherapy in metastatic disease	(Zheng et al., 2025)
	GX-188E	HPV16/18 DNA vaccine	Phase II	Enhanced T-cell responses	Requires combination therapy	(De Sousa et al., 2022)
Combination Therapy	ISA101 + Nivolumab	Vaccine + checkpoint blockade	Phase II	15.3 months median OS	Toxicity-related discontinuation	(Lorusso et al., 2025)
	ISA101 + Carboplatin-Paclitaxel	Vaccine + chemotherapy	Phase II	>50% tumor size reduction	Multiple immune barriers	(Liu et al., 2025)
Anti-angiogenic	Bevacizumab + Chemotherapy	VEGF inhibition	First-line approved	5-month OS extension	Severe anemia, neutropenia	(Menderes et al., 2016)
	Apatinib	VEGFR-2 inhibition	Retrospective studies	Delayed disease progression	Limited efficacy data	(Liu et al., 2025)
	Nintedanib	Multi-kinase inhibitor	Phase II	An extended OS in combination	Increased hematological toxicity	(Liu et al., 2025)
Metabolic Targeting	Caffeic acid + Metformin	Glutaminase/ME1 inhibition	Preclinical	Energy balance disruption	Limited to experimental studies	(Ling et al., 2022)
Anti-inflammatory	COX-2 inhibitors	Inflammatory mediator reduction	Investigational	Reduced chronic inflammation	Prognostic impact unclear	(Liu et al., 2025)

Non-steroidal anti-inflammatory drugs and COX-2 inhibitors can reduce the production of inflammatory mediators, thereby suppressing HPV-induced chronic inflammation. Studies indicate that the tyrosine kinase inhibitor sunitinib can increase nitric oxide synthase production and trigger apoptosis. Additionally, natural compounds, including resveratrol, catechins, curcumin, vitamin B6, and vitamin C, as well as other antioxidants, can promote the apoptosis of cervical cancer cells (Nadile et al., 2022). However, the prognostic impact of these antioxidants on cervical cancer patients requires further investigation.

### 5.1 Anti-angiogenic therapy

The Clinical Application Guidelines for Anti-Angiogenic Drugs in Cervical Cancer recommend bevacizumab combined with chemotherapy as first-line treatment for patients with recurrent, metastatic, and advanced cervical cancer (Li J. et al., 2025). Apatinib specifically inhibits VEGF receptor-2 tyrosine kinase activity, delaying cervical cancer progression (Xiao et al., 2020). In a randomized, double-blind phase II clinical study conducted by the European Network of Gynaecological Oncological Trial Groups (ENGOT) on patients with recurrent or treatment-naive advanced (stage IVB) cervical cancer, overall survival was extended by 5 months in patients receiving carboplatin plus paclitaxel chemotherapy combined with oral nintedanib 200 mg (Vergote et al., 2023). However, nintedanib is associated with more severe anemia and neutropenia. While anti-angiogenic drugs offer alternative treatment options for advanced cervical cancer, the efficacy of combination regimens, optimal therapeutic protocols, and predictive biomarkers for combination therapy efficacy requires further investigation.

## 6 Enhanced HPV vaccine efficacy through multi-agent therapeutic strategies

**Figure 3:**
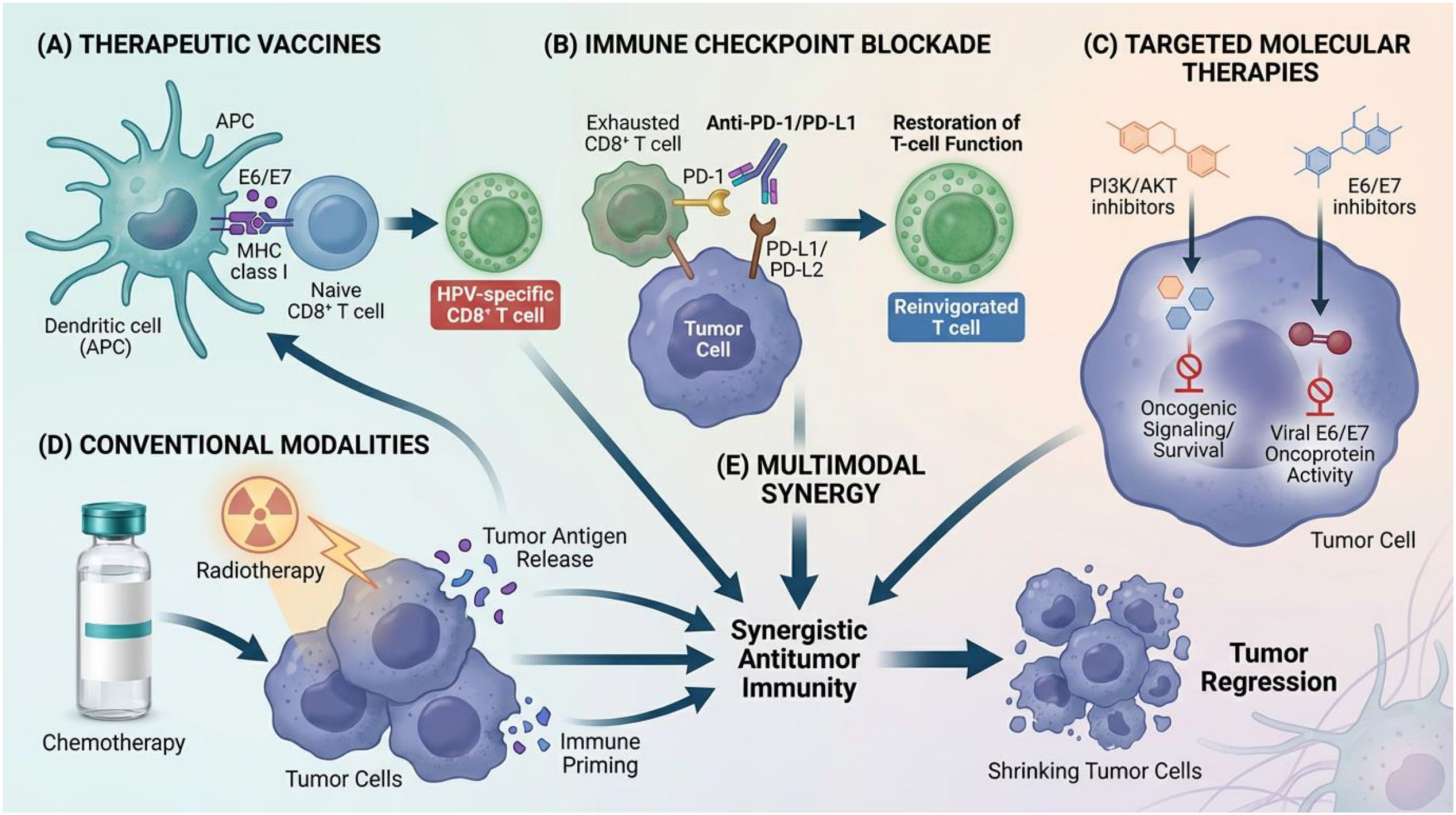
Multimodal therapeutic strategies targeting HPV-associated cervical cancer.

For HPV-driven cervical cancer, there is clear clinical evidence indicating that monotherapy based on E6 and E7 immune platforms is ineffective. To overcome these clinical limitations, a multimodal treatment approach combining vaccines with complementary tumor drugs (such as immune checkpoint modulators and cytotoxic chemotherapy) has enhanced therapeutic efficacy through a synergistic anti-tumor mechanism (**Figure 3**). The study evaluating the combination of pembrolizumab with HPV E6/E7 DNA immunization (GX-188E) provides a clinical example of this approach. These studies have shown that immune-mediated tumor suppression is enhanced in populations with advanced or recurrent cervical cancer while maintaining acceptable safety parameters (Youn et al., 2020). In the Phase II clinical evaluation of ISA101 combined with immune checkpoint inhibition, particularly navolizumab (anti-PD-1), HPV-16-positive cervical cancer patients exhibited an enhanced anti-tumor response of HPV-16-specific T lymphocytes and a weakened immunosuppressive mechanism within the tumor microenvironment (TME). Elevated pancreatic lipase and liver enzymes, fever, injection-site inflammation, nausea, and weakness are documented side effects of treatment (Glisson et al., 2017). Among patients with HPV-16-positive cervical cancer, the objective clinical response rate of ISA101-nivolumab combination therapy was 33%. The treatment plan includes the simultaneous administration of 100 μg of ISA101 on days 1, 22, and 50, and 3mg/kg of nivolumab every two weeks, starting from day 8, for 12 months. The three-year longitudinal data confirmed the effectiveness of continuous treatment, with a median overall survival of 15.3 months. Immunoidentification revealed a significant increase in the infiltration of CD3+CD8+PD-1+ cytotoxic T cells and CD68+ PD-L1-negative and CD68+ PD-L1-positive macrophages within the tumor. These immune changes are directly related to clinical outcomes. Additionally, a strong correlation exists between interferon-γ-related transcriptional patterns and therapeutic responses (van Poelgeest et al., 2016). However, due to treatment-related toxicity, some individuals had to discontinue nivolumab (Massarelli et al., 2019). ISA101, administered as monotherapy, demonstrated therapeutic efficacy in HPV16-positive high-grade vulvar intraepithelial neoplasia by activating antigen-specific T cells, leading to lesion clearance in vaccinated individuals (Melief et al., 2020). On the other hand, ISA101 did not produce strong antigen-specific T cell activation in patients with HPV16-related metastatic cervical cancer. The immunophenotype of peripheral blood mononuclear cells (PBMCs) revealed impaired antigen-presenting cell stimulation function, resulting in insufficient T lymphocyte activation. In addition, the increase in the bone marrow cell population promotes local immunosuppression. The immune cell profiles of these patients returned to normal after receiving chemotherapy with carboplatin and paclitaxel (Burd, 2003).

From a mechanistic perspective, local application of imiquimod at the vaccination site may enhance CD8+ T cell responses in unresponsive individuals, thereby improving the vaccine’s performance. However, clinical data show that imiquimod does not significantly enhance vaccine efficacy. Therefore, the combined use of ISA101 with other immunomodulatory drugs is expected to enhance the therapeutic effect. However, further clinical research is still needed to determine the optimal joint program (Ding et al., 2022).

## 7 Conclusion and future direction

This review has outlined how impaired PRR-mediated sensing, HPV-driven immune evasion, and progressive microenvironmental reprogramming collectively shape cervical carcinogenesis. Innate immune dysregulation is a defining feature of HPV-induced cervical carcinogenesis, in which viral oncoproteins strategically disable PRR-mediated antiviral responses, disrupt interferon signaling, alter epithelial-stromal communication, and establish an immunosuppressive microenvironment that favors persistent infection and malignant transformation. The convergence of impaired antigen presentation, regulatory immune cell recruitment, metabolic reprogramming, angiogenesis, and chronic inflammation reveals how HPV reshapes the cervical immune landscape, enabling it to escape immune clearance. Continued progress in dissecting these innate immune pathways will be essential for identifying biomarkers of early immune tolerance disruption and for developing targeted interventions to restore antiviral immunity. Future work should explore PRR-specific therapeutic modulation, the integration of microbiome–immune interactions, the role of extracellular vesicles and inflammasomes in shaping the PIM, and the translational potential of combining vaccines, checkpoint inhibitors, metabolic therapies, and anti-angiogenic agents. A deeper mechanistic understanding of HPV innate immunity crosstalk will drive the next generation of preventive and therapeutic strategies aimed at improving clinical outcomes and reducing the global burden of HPV-associated cervical cancer.
